# Decline in maternal death due to obstetric haemorrhage between 2010 and 2017 in Japan

**DOI:** 10.1038/s41598-019-47378-z

**Published:** 2019-07-30

**Authors:** Junichi Hasegawa, Shinji Katsuragi, Hiroaki Tanaka, Akiko Kurasaki, Masamitsu Nakamura, Takeshi Murakoshi, Masahiko Nakata, Naohiro Kanayama, Akihiko Sekizawa, Ishiwata Isamu, Katsuyuki Kinoshita, Tomoaki Ikeda

**Affiliations:** 1 Department of Obstetrics and Gynecology, St. Marianna University School of Medicine, Kanagawa, Japan; 2 Department of Obstetrics and Gynecology, Sakakibara Heart Institute, Tokyo, Japan; 3 Department of Obstetrics and Gynecology, Mie University School of Medicine, Mie, Japan; 4 Department of Obstetrics and Gynecology, Showa University School of Medicine, Tokyo, Japan; 5 Division of Perinatology, Maternal and Perinatal Care Center, Seirei Hamamatsu General Hospital, Hamamatsu, Japan; 6 Department of Obstetrics and Gynecology, Toho University School of Medicine, Tokyo, Japan; 7 Department of Obstetrics and Gynaecology, Hamamatsu University School of Medicine, Hamamatsu, Japan; 8 Ishiwata Obstetrics and Gynecology Hospital, Ibaraki, Japan; 9 Seijyo Kinoshita Hospital, Tokyo, Japan

**Keywords:** Epidemiology, Preventive medicine

## Abstract

This descriptive study was based on the maternal death registration system established by the Japan Association of Obstetricians and Gynecologists and the Maternal Death Exploratory Committee (JMDEC). 361 women died during pregnancy or within 42 days after delivery between January 2010 and June 2017 throughout Japan were analysed, in order to investigate the trend in maternal deaths related to obstetric medical practice. Reports of maternal death were consistent, ranging from 45 cases in 2010 to 44 cases in 2017. Among all maternal deaths, the frequency of deaths due to obstetric haemorrhage ranged from 29% (2010) to 7% (2017) (p < 0.001). The causes of obstetric haemorrhage have progressively reduced, especially maternal deaths due to uterine inversion and laceration have not occurred since 2014. The remaining causes of obstetric haemorrhage-related maternal deaths were placenta accreta spectrum, placental abruption, and severe forms of uterine focused amniotic fluid embolism. We believe the activities of the JMDEC including annual recommendations and simulation programs are improving the medical practices of obstetric care providers in Japan, resulting in a reduction of maternal deaths due to obstetric haemorrhage.

## Introduction

The Japan Association of Obstetricians and Gynecologists (JAOG) established a registration system for maternal deaths and the Japan Maternal Death Exploratory Committee (JMDEC) in 2010 to improve the quality of obstetric healthcare and ultimately prevent maternal deaths.

**Figure 1:**
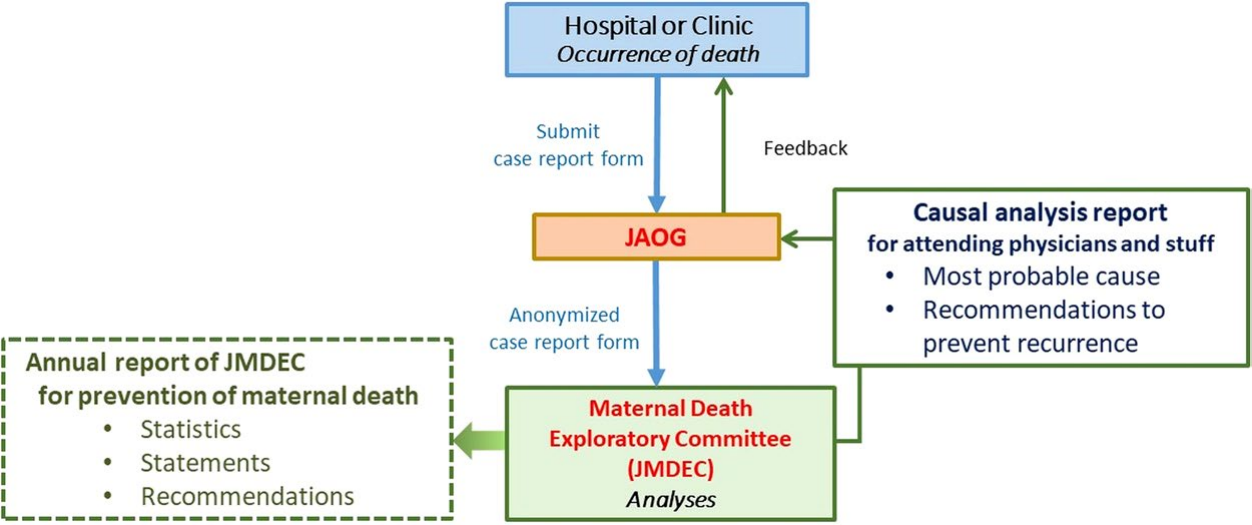
Maternal death registration system in Japan.

An outline of this registration system is demonstrated in Fig. 1. If maternal death occurs, attending physicians submit detailed report forms to the JAOG with medical records, including the anaesthetic record, medical images, laboratory data, and pathological and autopsy report. After anonymisation of report forms by JAOG, all forms and medical records throughout Japan are sent to JMDEC and the individual data are analysed for factors associated with maternal mortality and the circumstances of death by the JMDEC which comprises more than 20 specialists who attend review sessions every month^1^. After deep discussion, a causal analysis report on each case of maternal death, including the most probable cause and recommendations, is made and sent back to JAOG^1^. Finally, the report is provided to the submitted institution from JAOG. Causal analysis report is provided only to the attending physicians and staff to be used only within the facility that encountered maternal death, to improve their daily clinical behaviour and circumstance, providing objective opinions from specialists^1^. Since causal analysis reports are not open, maternal deaths are completely registered and analysed by JMDEC and are the same as the number of maternal deaths reported by the Ministry of Health, Labour and Welfare, Japan^2^.

JMDEC also makes a booklet on “Annual report of JMDEC; Recommendations for saving mothers” with comprehensive statistics, statements and, recommendations, based on monthly discussions and analyses. These are sent to obstetric care providers throughout Japan, regardless of the experience of maternal death. In the first year analysis after establishing the committee, it was revealed that the deaths associated with obstetric haemorrhage was the most frequent cause, similar to the 20^th^ century, in Japan^3^. There are 2500 obstetric facilities providing delivery services throughout Japan for approximately one million deliveries per year, and more than half of all deliveries are managed in private clinics operated by one, or sometimes, two obstetricians. Since pregnant women are likely to prioritize accessibility and comfort of the delivery facilities, they prefer such private facilities. Consequently, half of maternal deaths were among women who developed a complication at a private facility and were transported to tertiary hospitals.

Furthermore, approximately half of maternal deaths due to obstetric haemorrhage were considered to be potentially preventable if awareness of vital sings, the initial treatment, the timing of maternal transport, and intra- or inter-hospital relations could be improved^1^. The JMDEC had already provided a strong statement that appropriate primary care and immediate transport to the intensive care are required in cases of maternal emergencies in lectures and the booklet of annual recommendations.

Since we hypothesised that activities of the JMDEC might improve medical practices of obstetric care providers in Japan leading to a reduction in maternal deaths, we investigated the last 8-year trend in the magnitude of maternal mortality and causes of maternal death related to obstetric medical practice in Japan.

## Patients and Methods

A descriptive study was performed to investigate the last 8-year trend in Japan in causes of maternal death.

### Review and analysis of maternal deaths

The patients were women who died during pregnancy or within 42 days after delivery between 2010 and 2017 and analysed by JMDEC throughout Japan. The JMDEC consists of 15 obstetricians, 4 anaesthesiologists, 2 emergency physicians, 2 pathologists, and some specialists and forensics experts^1^. The registration system is the same as described in our previous reports^1,4–6^.

When maternal death occurs, attending physician submits the case of maternal death to the registration system of the JAOG. They send a report form to JAOG. The report form consists of 22 pages with approximately 100 questions which aim to elicit detailed information about the clinical history, the facility characteristics, and the personnel who participated in the patient’s care, along with the medical records, including the anaesthetic record, medical images, laboratory data, and pathological and autopsy reports^1^.

All individual information in the report form and medical records, including maternal characteristics, clinicians, and region of the facility are anonymised in JAOG. All these anonymised data throughout Japan are sent to JMDEC, and analysed and discussed for factors associated with maternal mortality and the circumstances of death in the JMDEC monthly meeting^1^.

Finally, after deep discussion, the JMDEC make a causal analysis report on each case of maternal death, including the most probable cause and recommendations, and send it back to JAOG. Then, causal analysis report is sent back via JAOG to the obstetric care provider at which the death occurred^1^ (Fig. 1).

### Classification and definitions

Classification of maternal deaths in the present study was based on using the globally used 10th edition of the International Statistical Classification of Diseases and Related Health Problems (ICD) codes and ICD-maternal mortality (ICD-MM) provided by the World Health Organization (WHO)^7,8^. Late maternal deaths, which was defined by the death of a woman from direct or indirect obstetrical causes in more than 42 days, but less than one year, after termination of pregnancy were excluded.

#### Amniotic fluid embolism (AFE)

Amniotic fluid embolism (O88.1) is classified in obstetric embolism in ICD-MM. This is an anaphylactoid syndrome of pregnancy. AFE was defined based on the Japan consensus criteria which is based on the US/UK criteria^9^.

#### Obstetric haemorrhage

Obstetric haemorrhage was included like the diseases described in Group 3: obstetric haemorrhage in ICD-MM^8^. Besides, we included uterine focused AFE (Uterine type AFE) in this category, when uterine atony, without other criteria of obstetric haemorrhage, could be observed macroscopically with a large oedematous uterus resulting in the rapid development of DIC, and foetal debris and amniotic fluid components were found in the removed uterus histologically. It is considered that the local flow of amniotic fluid into uterine tissues cause an anaphylactoid reaction in the uterus^9^.

### Statistical analyses

Statistical analyses were performed using the Statistical Package for Social Science (SPSS) software program (Windows version 20.0 J; Chicago, IL, USA).

### Ethics statement

This study was approved by the ethics board of National Cerebral and Cardiovascular Center, Osaka, Japan and the JAOG. This investigation was conducted in accordance with the principles of the Declaration of Helsinki. Informed consent was not obtained from patients or their families, because this study was based on the analysis of institutional forms, and the patient records/information was anonymised prior to the analysis.

## Results

There were 361 maternal deaths, as defined by the death of a woman from direct or indirect obstetrical causes in less than 42 days after termination of pregnancy between January 2010 and June 2017 throughout Japan. Their characteristics are demonstrated in Table 1. Regarding the location of initial symptoms associated with maternal death, 35% was in obstetric hospitals, private clinics, or midwifery homes, in which medical resources of intensive treatment for maternal emergency is limited; thus, a half of the patients who died had needed maternal transport to tertiary hospitals. Initial cardio-pulmonary arrest occurred in 22% of the patients at a clinic or obstetric hospital before maternal transfer or during transfer in an ambulance.

**Table 1:** Characteristics of the patients (n = 361).

**Maternal characteristics**	
Maternal age, y (median, range)	34 (19–46)
Gravida, n (median, range)	1 (0–9)
Parity, n (median, range)	1 (0–8)
Nulliparous	50% (181)
**Delivery**	
Normal spontaneous delivery	22% (79)
With uterine fundal pressure	2% (8)
Instrumental delivery	12% (43)
Caesarean section	36% (129)
Before delivery	18% (64)
Epidural analgesia for labour	6% (20)
**Timing of onset**	
During pregnancy	41% (148)
During labour	16% (59)
During Caesarean section	5% (17)
Puerperium period	35% (125)
Others	3% (12)
**Location of onset**	
General hospital	29% (103)
Obstetric hospital	9% (32)
Private clinic	25% (90)
Midwifery home	1% (3)
Outside of medical institution	37% (133)
**Maternal transport to tertiary hospital**	51% (184)
**Location of initial cardio-pulmonary arrest**	
General hospital	59% (214)
Obstetric hospital	4% (13)
Clinic before maternal transfer or in ambulance	22% (79)
Outside of medical institution	15% (55)
**Autopsy**	
Performed	36% (131)
Pathological autopsy, n	82
Administrative autopsy, n	10
Judicial autopsy, n	39
Not performed	64% (230)

Data represented in number (percentage), median (range).

**Figure 2:**
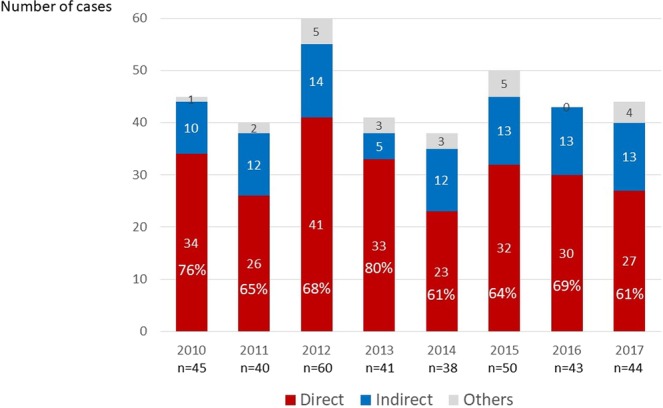
Recent trend in incidence of direct and indirect maternal deaths in Japan.

The recent trend in the frequency of direct maternal deaths in Japan is demonstrated in Fig. 2. The reports of maternal death were consistent, from 45 cases in 2010 to 44 cases in 2017. The incidences of direct obstetric deaths were 76% and 61% in 2010 and 2017, respectively.

**Figure 3:**
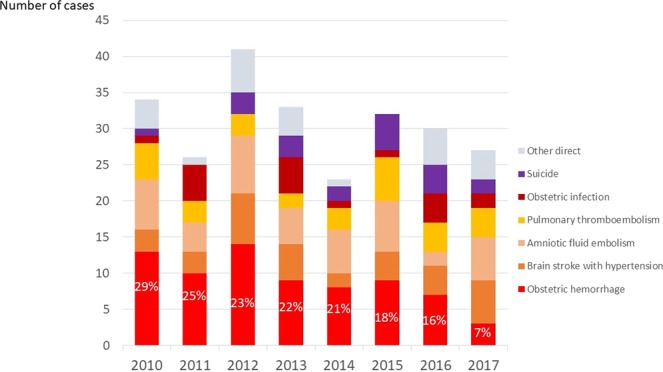
Recent trend in direct maternal deaths stratified by causes in Japan.

The recent trend in direct maternal deaths stratified by causes in Japan is demonstrated in Fig. 3. Among all maternal deaths, the frequency of deaths due to obstetric haemorrhage reduced from 29% in 2010 to 7% in 2017 (p < 0.001), while the frequency of the maternal deaths due to the other causes were consistent.

**Figure 4:**
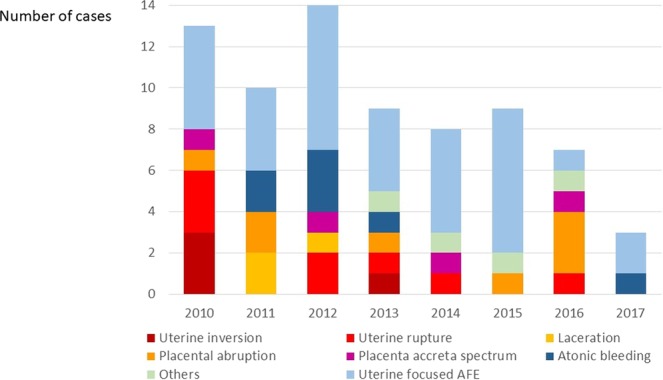
Recent trend in obstetric haemorrhage-related maternal deaths stratified by causes in Japan.

The recent trend in obstetric haemorrhage-related maternal deaths stratified by causes in Japan is demonstrated in Fig. 4. Notably, each cause of maternal death has since reduced, especially maternal deaths due to uterine inversion and laceration have not occurred since 2014. On the other hand, the remaining causes of obstetric haemorrhage-related maternal deaths were PAS, placental abruption, and severe forms of uterine focused AFE.

The case series of maternal deaths associated with placental abruption are shown in Table 2. Out of eight patients, one woman, who had not reported for pregnancy check-ups, died at home. Only one had complications with hypertensive disorder, but the others had low-risk pregnancy, complicated with placental abruption, rapidly resulting in intrauterine foetal death (IUFD). The initial symptoms were mainly abdominal pain, rather than external bleeding. In the cases where the serum fibrinogen concentration was measured, the initial measurement values were below 100 mg/dl.

**Table 2:** Maternal deaths associated with placental abruption.

Case	Parity	GW	Hypertension	Symptom	IUFD	Mode of delivery	Maternal transport between hospitals	Serum fibrinogen concentration	Problem
1	1	35	−	Unknown	+	not delivery	Death at home	n/r	without pregnancy check-ups
2	1	40	−	abdominal pain	−	transvaginal	+	n/r	delay transport
3	1	37	−	abdominal pain	+	CS	−	50 mg/dl	delay blood infusion
4	0	38	+	abdominal pain	+	transvaginal	−	n/r	delay blood infusion and haemostasis
5	1	34	−	abdominal pain	+	transvaginal	−	62 mg/dl	delay blood infusion and haemostasis
6	1	27	−	abdominal pain	+	transvaginal	+	25 mg/dl	delay FFP infusion
7	0	31	−	abdominal pain	+	transvaginal	+	67 mg/dl	delay FFP infusion
8	0	36	−	abdominal pain	+	CS	+	n/r	delay transport

GW; gestational weeks, IUFD; intrauterine foetal death, FFP; fresh frozen plasma.

## Discussion

The current maternal mortality rate in Japan, which is estimated to be around 4 in 100,000 deliveries^10^, is similar to that in other developed countries. Obstetric haemorrhage has always been the most frequent cause of maternal deaths in Japan. However, the frequency of maternal deaths due to obstetric haemorrhage rapidly declined in the last 8 years; as a result, the maternal death rate reached 61% in 2017. In a WHO systematic analysis of global causes of maternal death, about 73% of all maternal deaths between 2003 and 2009 were due to direct obstetric causes^11^; particularly for obstetric haemorrhage, while the rates were 16.3% and 27.1% in developed and developing regions, respectively^11^. The proportion of causes of maternal deaths in Japan appears to be close to that in developed countries.

We believe that the decline in obstetric haemorrhage-related maternal death rate can be explained by improvement of management provided by obstetric caregivers throughout Japan, rather than by medical centralization of obstetric facilities. Not only did the frequency of all deliveries in private clinics remain almost the same from 48% in 2010 to 46% in 2017^10^, but proportion of locations, whether hospital or private clinic when initial symptom associated with maternal death occurred, was also consistent in the 8 years of our study^12^. We think activities of the JMDEC are beneficial to obstetric caregivers year by year.

In fact, in our analyses of cases, in the earlier years, maternal deaths resulting from deteriorating continuous small bleeding, which was expected to be managed over an extended time period, were frequently reported. However, such reports of several problems in the diagnostic procedures, treatment strategies, and the inter-hospital and intra-hospital systems were likely reduced in the recent years. There seems to be an improvement in the timing of the diagnosis and treatment, including maternal transport, intervention, and blood transfusion and their systems.

**Figure 5:**
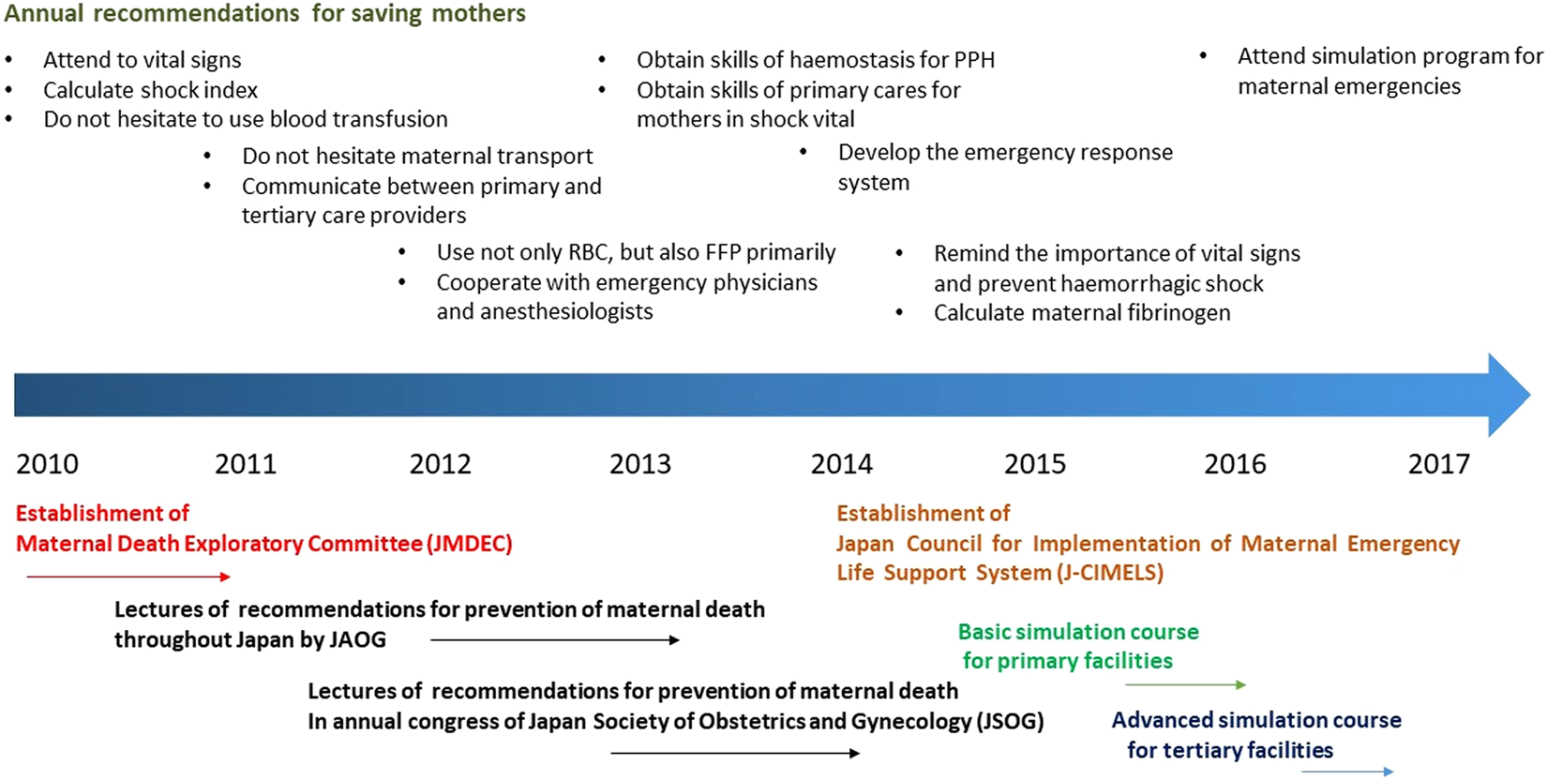
Activities of Maternal Death Exploratory Committee in Japan.

The activities of JMDEC are demonstrated in Fig. 5. One of the main activities of JMDEC is the publication of a booklet on “Annual report of JMDEC for prevention of maternal death” and lectures providing strong statements for maternal emergencies from 2010. Besides, the Committee had also developed a maternal emergency simulation program for perinatal caregivers to reduce maternal morbidity and mortality, with related academic societies, such as anaesthesiology and emergency medicine. Consequently, a maternal emergency simulation program for doctors, nurses, and midwives (J-MELS; Japan Maternal Emergency Life-saving course) has been established since 2015. The curriculum in this course includes immediate interpretation and primary care for maternal emergency, decision of maternal transport, and basic life support. J-MELS has already been provided throughout Japan, in which more than 10000 obstetric caregivers have participated. We believe that these improvements are due to the effects of our annual recommendations, widely progressive simulation programs, and self-improvement of their daily clinical management.

However, the JMDEC still considers that a half of maternal deaths due to haemorrhage are not preventable, even if appropriate management or intensive care in the tertiary hospital is provided^1^. On the other hand, we considered that improved management of some obstetric haemorrhagic conditions, including PAS and placental abruption, can prevent further maternal deaths. In cases with PAS, including suspected PAS during retained placenta, the underestimation of blood loss count and delayed blood transfusion, unskilled surgeons, and non-strategic surgeries for PAS were noted even in the treatment at the tertiary hospitals. If the surgeon lacks confidence in performing these procedures or rapid blood transfusion is unavailable, the patient should be precedingly referred to an appropriate hospital^13^.

Besides, maternal deaths after placental abruption with IUFD, which presented with abdominal pain prior to external bleeding, were noticeable. Namely, these are less common concealed types of placental abruption, which are likely to be concomitant with severe consumption of coagulation factors^14^. Since concealed abruption occurs when blood accumulates behind the placenta, bleeding may occur into the uterine myometrium, leading to a beefy boggy uterus, called a Couvelaire uterus^14^. Then, haemorrhage in the myometrium leads to uterine atony and further consumption of coagulation factors, and abruption involving the entire placenta leads to IUFD. Although obstetricians are unlikely to decide caesarean section or maternal transfer to tertiary hospital immediately when diagnosis of IUFD, obstetric caregivers should keep in mind that concealed abruption and IUFD due to placental abruption are the most serious maternal conditions and immediate interventions are required.

The JMDEC considers uterine focused AFE, which usually results in the rapid development of consumptive coagulopathy/DIC, as one of the limitations to reduce further maternal death due to obstetric haemorrhage. A uterine focused AFE occurs when foetal debris and amniotic fluid components are found in the uterus in the pathological examination of cases of severe uterine haemorrhage, like atonic bleeding, in the absence of other obstetric haemorrhagic complications^1^. There are no specific medications for such severe haemorrhage; hysterectomy for haemostasis with massive blood transfusion is required. It is reported that blood transfusion with fresh frozen plasma (red blood cell ratio of 1 or more) was more effective with a higher survival rate^15^. However, since blood transfusion is usually not available in private clinics, immediate maternal transfer to a tertiary hospital is better management in such situations in Japan.

## Conclusion

Among all maternal deaths in Japan, the causes of obstetric haemorrhage have progressively reduced from 29% to 7% for 8 years. We believe the activities of the JMDEC including annual recommendations and simulation programs are improving the medical practices of obstetric care providers in Japan, leading in a reduction of maternal deaths due to obstetric haemorrhage. Simulation program (J-MELS) is also strongly encouraging caregivers to pay attention to vital signs and improve the primary management of obstetric haemorrhage in daily clinical settings. For further reduction of maternal death, a greater focus on preventing and treating indirect causes of death is also needed.
